# Dramatic Increase in Expression of a Transgene by Insertion of Promoters Downstream of the Cargo Gene

**DOI:** 10.1007/s12033-014-9738-0

**Published:** 2014-02-14

**Authors:** Masakiyo Sakaguchi, Masami Watanabe, Rie Kinoshita, Haruki Kaku, Hideo Ueki, Junichiro Futami, Hitoshi Murata, Yusuke Inoue, Shun-Ai Li, Peng Huang, Endy Widya Putranto, I. Made Winarsa Ruma, Yasutomo Nasu, Hiromi Kumon, Nam-ho Huh

**Affiliations:** 1 Department of Cell Biology, Graduate School of Medicine, Dentistry and Pharmaceutical Sciences, Okayama University, Okayama, 700-8558 Japan; 2 Center for Innovative Clinical Medicine, Okayama University Hospital, Okayama, 700-8558 Japan; 3 Department of Urology, Graduate School of Medicine, Dentistry and Pharmaceutical Sciences, Okayama University, Okayama, Japan; 4 Department of Medical and Bioengineering Science, Graduate School of Natural Science and Technology, Okayama University, Okayama, 700-8558 Japan; 5 Department of Chemistry and Chemical Biology, Graduate School of Engineering, Gunma University, 1-5-1 Tenjin-cho, Kiryu, Gunma 376-8515 Japan

**Keywords:** Gene expression, Plasmid, Adenovirus, Recombinant protein, Gene therapy

## Abstract

For expression of genes in mammalian cells, various vectors have been developed using promoters including CMV, EF-1α, and CAG promoters and have been widely used. However, such expression vectors sometimes fail to attain sufficient expression levels depending on the nature of cargo genes and/or on host cell types. In the present study, we aimed to develop a potent promoter system that enables high expression levels of cargo genes ubiquitously in many different cell types. We found that insertion of an additional promoter downstream of a cargo gene greatly enhanced the expression levels. Among the constructs we tested, C-TSC cassette (C: CMV-RU5′ located upstream; TSC: another promoter unit composed of triple tandem promoters, hTERT, SV40, and CMV, located downstream of the cDNA plus a polyadenylation signal) had the most potent capability, showing far higher efficiency than that of potent conventional vector systems. The results indicate that the new expression system is useful for production of recombinant proteins in mammalian cells and for application as a gene therapeutic measure.

## Introduction

In many biomedical studies, high-level expression of a gene in mammalian cells is a prime issue. High-level expression of a gene is necessary for assessment of biological effects of the gene, application as a therapeutic measure in a cell-autonomous manner, and therapeutic approaches using recombinant proteins produced under economical large-scale conditions. A variety of strong promoters have been exploited with the aim of high-level gene expression. Promoters of viral origin have been most widely used for this purpose. Cytomegalovirus (CMV) [1] and SV40 [2, 3] promoters are among the strongest promoters in a wide variety of cell types derived from different species. Promoters of mammalian origin have also been utilized. Promoters of human elongation factor-1 alpha (EF-1α) [4] and β-actin [5, 6] are constitutively active in a broad range of cell types. Both promoters are often active in cells in which viral promoters fail to express downstream genes and also in cells in which the viral promoters are gradually silenced as observed in embryonic stem cells [7].

To further enhance the function of promoters, various enhancer elements have been added upstream of the promoters. A representative example is the CAG promoter, a combination of the CMV immediate-early enhancer and a modified chicken β-actin promoter [8]. The CAG promoter has frequently been used to drive high-level gene expression in mammalian cells. The R segment and a part of the U5 sequence (R-U5′) of the HTLV type 1 long terminal repeat (LTR) were inserted downstream of viral and mammalian promoters [9]. The modification resulted in not only enhancement of steady-state transcription but also elevated translation efficiency through mRNA stabilization [9]. Although appropriate combinations of enhancer and promoter elements have thus showed efficient gene expression capability and greatly contributed to biomedical science, such expression vectors sometimes fail to attain sufficient protein expression levels depending on the nature of cargo genes and/or on host cell types. Many different mechanisms are potentially involved in determining the final stationary protein levels. The secondary structure of the cargo mRNA gene plays a critical role in the bio-synthesis of proteins, especially during translation process [10]. The stability of a newly synthesized protein also contributes to the expression level of the cargo gene. In addition, regulatory factors that control promoter elements of the vector are expressed in host cells with different protein levels.

In the present study, we, therefore, attempted to develop a potent promoter system that enables high-level expression of cargo genes ubiquitously in different cell types. After various trials, we eventually found that insertion of an additional promoter downstream of a cargo gene greatly enhanced the expression levels. Among the constructs we tested, C-TSC cassette (C: CMV-RU5′ located upstream; TCS: another promoter unit composed of triple tandem promoters, hTERT, SV40 and CMV, located downstream of the cDNA plus a polyadenylation signal) had the most potent capability, showing far higher efficiency than that of potent conventional vector systems. The data indicate that the new expression system is useful for production of recombinant proteins in mammalian cells and for application as a gene therapeutic measure.

## Materials and Methods

### Cells and Cell Culture

The following human cell lines were used: HEK293 (embryonic kidney cell line; ATCC, Rockville, MD), MCF7 (mammary gland adenocarcinoma cell line; ATCC), PC-3 (prostate adenocarcinoma cell line; ATCC), HeLa (cervix adenocarcinoma cell line; ATCC), HepG2 (hepatocellular carcinoma cell line; ATCC), and KPK-1 (renal clear cell carcinoma cell line; Clonetics, San Diego, California). These cell lines were cultivated in D/F medium (Invitrogen, Carlsbad, CA) supplemented with 10 % FBS.

### Preparation of Plasmid Vectors

**Fig. 1:**
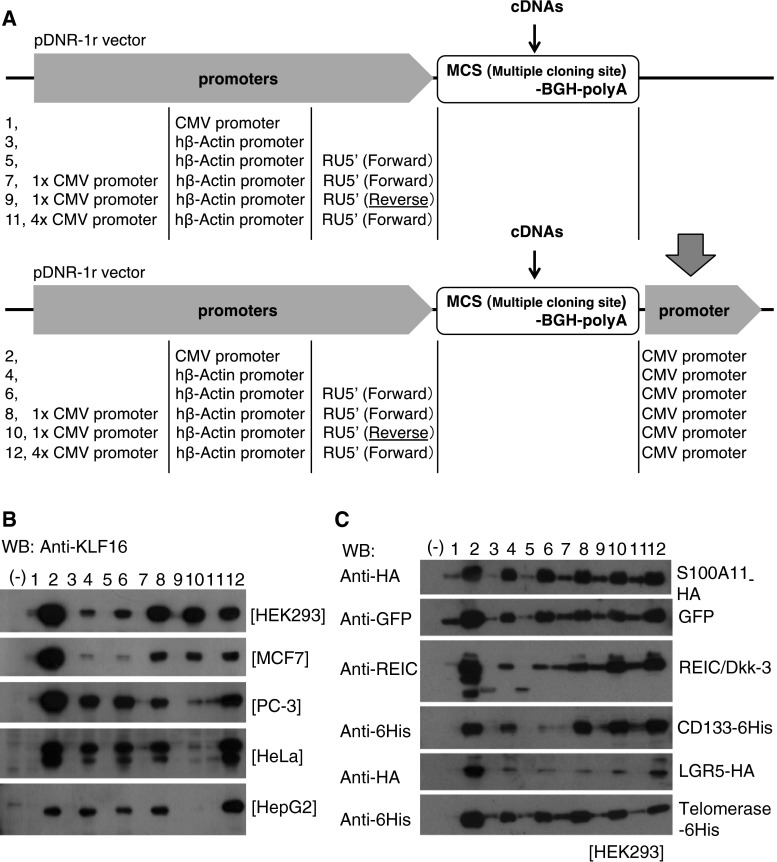
Schematic diagram of modified gene expression systems and their capabilities for gene expressions. **a** A series of indicated plasmids were constructed on the basis of the promoter-less pDNR-1r vector. **b** Expression of KLF16 protein was assessed by Western blot analysis after transfecting the indicated plasmids carrying KLF16 cDNA in HEK293, MCF7, PC-3, HeLa, and HepG2 cells. **c** Plasmid vectors carrying various cDNAs were constructed using the same series of vectors as those shown in (**A**). The vectors were transfected to HEK293 cells, and the level of each protein was determined by Western blot analysis. *Lane numbers* in **b** and **c** correspond to the vector numbers shown in (**a**)

The newly constructed expression cassettes are shown in Fig. 1a. The promoters, RU5′, BGH (bovine growth hormone) polyadenylation (polyA) signal, and a sequence for multiple cloning sites, were synthesized by IDT Inc. (Coralville, IA) and inserted into pDNR-1r promoter-less vector (Clontech, Mountain View, CA) or pIDT-SMART promoter-less vector (IDT Inc.). The RU5′ sequence (269 bp: Accession No. J02029 (374–642)) is derived from the R segment and a part of the U5 sequence of HTLV Type 1 long terminal repeat and used to enhance transcription efficiency [9]. Sequences of the promoter elements were as follows: hTERT (189 bp: Accession No. DQ264729 (1618–1806)), SV40 (319 bp: Accession No. AY864928 (2156–2474)), and CMV (479 bp: Accession No. AJ318513 (159–637)). The CAG promoter was obtained from the pCAGGS vector (a kind gift from Dr. Jun-ichi Miyazaki; Osaka University, Japan). pTracer-EF/V5-His-A and pEF6/Myc-His-A were purchased from Invitrogen. Full-length cDNAs of human S100A11, REIC/Dkk-3, CD133, LGR5 (leucine-rich repeat-containing G protein-coupled receptor 5), telomerase, erythropoietin (EPO), and green fluorescence protein (GFP) were amplified by RT-PCR.

### Construction and Production of Adenovirus Vectors

REIC/Dkk3 cDNA was cloned into a pShuttle plasmid vector driven by the CMV promoter (Clontech) followed by insertion of the cassette consisting of the BGH poly A signal and triple tandem promoters of hTERT, SV40, and CMV. The resulting donor plasmid was used for constructing an adenovirus vector according to the manufacturer’s instructions.

### Transfection

Cells were transiently transfected with plasmid vectors using FuGENE-HD (Promega BioSciences, San Luis Obispo, CA), Trans IT-keratinocyte transfection reagent (Mirus, Madison, WI), and Lipofectamine 2,000 (Invitrogen). Adenovirus vectors were infected at the indicated MOI (multiplicity of infection) as previously described [11].

### Purification of EPO

Human erythropoietin tagged with 6His at the C-terminal side (EPO-6His) was expressed using the FreeStyle 293 Expression System (Invitrogen), which enables recovery of EPO secreted into the medium of 293-F cells cultured in suspension according to the manufacturer’s instructions. EPO-6His in the culture medium was purified using Co^2+^-resin column chromatography (GE Healthcare Life Sciences, Tokyo, Japan) and quantitated by Bradford protein assay method.

### Western Blot Analysis

Cells were harvested 24 h after transfection or infection with expression vectors and subjected to SDS-PAGE and Western blotting under the conditions described previously [11]. A rabbit anti-human REIC/Dkk-3 antibody was raised in our laboratory [11]. Goat anti-human KLF16 antibody (Abcam, Inc., Cambridge, MA), anti-6x histidine antibody (MBL Co., Nagoya, Japan), anti-HA antibody (Cell Signaling Technology, Danvers, MA), and anti-GFP antibody (Clontech) were purchased.

### Transient transfection and quantitative RT-PCR

**Fig. 2:**
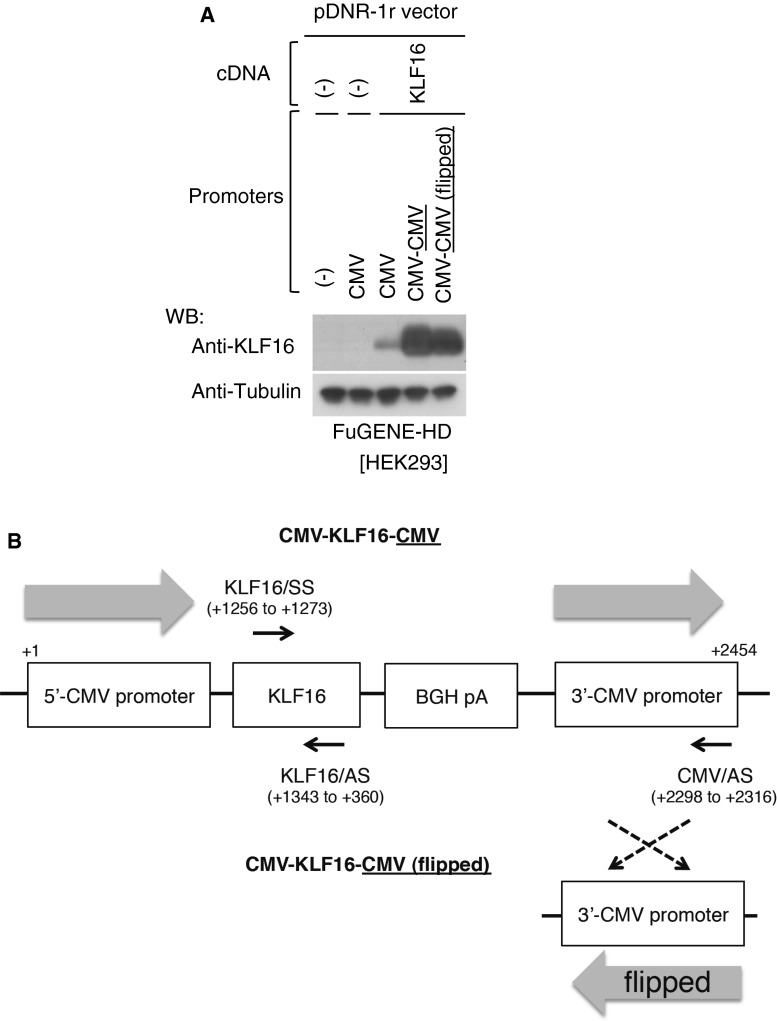
Orientation of the 3′-CMV promoter-independent gene expression. **a** Orientation of the 3′-CMV promoter in the CMV-KLF16-CMV construct was flipped, termed CMV-KLF16-CMV (flipped), and then expression of KLF16 protein was assessed by Western blot analysis after transfecting the plasmid including indicated comparative vectors in HEK293. Tubulin was used as a control for loaded amounts of protein. **b** Primers were designed to the indicated locations of the CMV-KLF16-CMV and the CMV-KLF16-CMV (flipped) constructs (see Materials and Methods)

The pDNR-1r-based cassettes, CMV–CMV or pCMV–KLF16–CMV, were transfected to HEK293 cells (2 × 10^4^) in 96-well plate with polyethylenimine Max (Polyscience, Warrington, PA) as a transfection reagent. After 48 h, the cells were washed with phosphate-buffered saline, and total RNA was extracted using Tripure Isolation Reagent (Roche, Tokyo, Japan). After removing contaminated DNA with TURBO DNA-free kit (Life technologies), reverse transcription was performed using ReverTra Ace qCR RT kit (TOYOBO, Osaka, Japan) with specific primers (Fig. 2b) for sense strand (CMV/AS; 5′-ATCCACGCCCATTGATGTA-3′) and anti-sense strand (KLF16/SS; 5′-CAGGGCTGCGACAAGAAG-3′) of KLF16 mRNA. Real-time PCR was performed using FastStart SYBR Green Master (Roche) with the specific primers (Fig. 2b) for detection of KLF16 (KLF16/SS and KLF16/AS; 5′-GAAGCGCTTGGAGCACAG-3′) on a LightCycler 480 system II (Roche). Copy number of the amplicons was estimated based on a standard curve made using a plasmid encoding KLF16 cDNA.

### Quantification of cytoplasmic and nuclear mRNA for KLF16

The pDNR-1r-based cassettes, CMV–CMV, CMV–KLF16–CMV, or CMV–KLF16, were co-transfected with pGL4.74 encoding Renilla luciferase (Promega) as an internal control to HEK293 cells (6 × 10^5^) in 6-cm dishes using polyethylenimine Max. After 36, 48, and 60 h, the cells were washed with phosphate-buffered saline and cytoplasmic, and nuclear RNA was extracted using Cytoplasmic & Nuclear RNA Purification Kit (Norgen Biotek, ON, Canada). Reverse transcription was performed using ReverTra Ace qPCR RT Master Mix with gDNA Remover (TOYOBO, Osaka, Japan) containing oligo dT and random hexamer primers. Real-time PCR was performed using the specific primers (Fig. 2b) for KLF16 (KLF16/SS and KLF16/AS primers). The levels of amplicons were expressed relative to Renilla Luciferase RNA using the ∆∆Ct method.

### In Vitro EPO Bioactivity

Bioactivity of purified EPO-6His was assayed by testing the proliferation dependence of the human erythroleukemic cell line TF-1 (CRL-2003, ATCC, Rockville, MD) [12]. The assay was performed in 96-well plates, and viable cells were estimated using an MTT reagent kit (Sigma, Saint Louis, MO) according to manufacturer procedures.

### Deglycosylation of EPO

Potential *N*-glycosylation of the purified EPO-6His was examined by digesting with peptide-*N*-glycosidase F (PNGase F; NEB, Ipswich, MA) in a solution indicated by the supplier.

### Apoptosis Assay

Twenty-four hours after inoculation, cells were infected with adenovirus vectors carrying REIC/Dkk3 at different MOIs and incubated for 72 h. Apoptotic cells were visualized by staining with Hoechst 33342 and analyzed as previously described [13]. Statistical significance in the difference was assessed by analysis of variance (ANOVA).

## Results

### Promoter(s) Placed at the 3′-side Remarkably Enhanced the Efficiency of Gene Expression

In our previous study in which we tried to express two cDNAs with respective promoters in one construct, we unexpectedly found that the presence of a promoter at the 3′-side of the cDNA significantly enhanced the efficiency of expression. To optimize the expression efficiency, we prepared various constructs as shown in Fig. 1a, choosing GC-rich KLF16 cDNA as a cargo that is empirically known to be extremely difficult in mammalian cells [14]. Vectors containing the CMV promoter, the human β-actin promoter, and the human β-actin promoter plus 1 or 4 CMV promoters did not show any appreciable expression (Fig. 1b). On the other hand, addition of the CMV promoter at the 3′-side of the cDNA dramatically enhanced the expression levels (Fig. 1b). The highest expression level was observed with a construct carrying one CMV promoter at each side (Fig. 1b, lane 2), indicating that the combination of 5′- and 3′-promoters is relevant.

We examined whether the 3′-CMV promoter is transcribed beyond the BGH polyA sequence. A quantitative RT-PCR analysis showed that a detectable amount (7.35 ± 3.25) of transcripts containing KLF16 flanked by the 3′-CMV promoter was observed when CMV/AS primer (Fig. 2b) was used for reverse transcription in cells transfected with the CMV–CMV vector (Table 1). On the other hand, a significant amount (400.19 ± 47.20) of transcripts containing KLF-16 without the 3′-CMV promoter was detected when KLF16/AS primer (Fig. 2b) was used for reverse transcription (Table 1). Subsequently, we also detected the anti-sense strand of KLF16 mRNA in cells transfected with the CMV–KLF16–CMV (Table 2). However, the amount (51.48 ± 7.48) was not significant when compared to sense transcripts of KLF16 (400.19 ± 47.20) in Table 1, suggesting that sense transcription in CMV–CMV vector is critically affected by the 3′-CMV promoter.

**Table 1:** Transcription of sense strand of 3′-CMV promoter in CMV–CMV vector

Vector	RT primer	copy number/ng RNA (×10^3^)
CMV–CMV	CMV/AS	ND
CMV–KLF16–CMV		7.35 ± 3.25
CMV–CMV	KLF16/AS	3.35 ± 0.17
CMV–KLF16–CMV		400.19 ± 47.20
CMV–CMV (flipped)		3.71 ± 0.34
CMV–KLF16–CMV (flipped)		337.34 ± 51.72

After transfection, copy number of sense strand of 3′-CMV promoter was measured. Data are mean ± SD.

*ND* not detected

**Table 2:** Transcription of anti-sense strand of KLF16 in CMV–CMV vector

Vector	RT primer	copy number/ng RNA (×10^3^)
CMV–CMV	KLF16/SS	0.29 ± 0.01
CMV–KLF16–CMV		51.48 ± 7.48
CMV–CMV (flipped)		0.38 ± 0.03
CMV–KLF16–CMV (flipped)		48.72 ± 5.35

After transfection, copy number of anti-sense strand of KLF16 mRNA was measured. Data are mean ± SD

To get an insight into the mechanisms for the high expression levels of proteins by the CMV–CMV vector, we examined relative amounts of mRNA in the cytoplasm and nuclei of HEK293 cells transfected with different vectors (Table 3). The ratio of the cytoplasmic (C) to nuclear (N) amounts of KLF16 mRNA was 0.74 (C/N) when expressed by the CMV–CMV vector, while that was 0.39 (C/N) by the 5′-CMV vector. This may be due to enhanced nuclear exportation and/or stabilization of cytoplasmic RNA when KLF16 was expressed in the CMV–CMV construct.

**Table 3:** KLF16 expression in cytoplasmic and nuclear fractions

Vector		36 h	48 h	60 h
CMV–CMV	C	1.00 ± 0.03	0.88 ± 0.02	0.75 ± 0.01
	N	1.52 ± 0.04	1.14 ± 0.03	1.43 ± 0.02
CMV–KLF16–CMV	C	723.95 ± 11.64	582.68 ± 13.89	638.99 ± 5.10
	N	905.86 ± 18.23	869.54 ± 42.46	854.96 ± 12.30
CMV–KLF16	C	266.84 ± 5.63	215.73 ± 3.76	257.72 ± 2.73
	N	611.56 ± 7.37	742.56 ± 8.88	616.27 ± 29.51

After transfection, relative expression of KLF16 mRNA was measured using C (cytoplasmic) and N (nuclear) fractions; cytoplasmic fraction. Data are mean ± SD

To exclude the possibility that the observed high efficiency was applicable only for KLF16, we examined various cDNAs, including S100A11 (a rather small protein of 11 kDa), GFP, REIC/Dkk3 (a secreted protein), CD133 (a transmembrane protein of 130 kDa), LGR5 (a transmembrane protein of 180 kDa), and telomerase (an intranuclear protein of 180 kDa), by inserting them into the same vector series as shown in Fig. 1a (Fig. 1c). Eventually, 3′-insertion of the CMV promoter resulted in elevated protein levels for the different cargo genes (Fig. 1c).

In order to gain more mechanistic insight, the orientation of the 3′-CMV promoter sequence in the CMV–KLF16–CMV construct was flipped and analyzed at both protein (Fig. 2a) and RNA expression levels (Table 1, 2). Based on the approach, we found that the efficient expression was almost unaffected by the reverse sequence of the 3′-CMV promoter at protein level (Fig. 2a). Similarly, transcripts of the sense strand of KLF16 were unaffected by the flipped 3′-CMV promoter (Table 1). In addition, significant transactivation of the anti-sense strand of KLF16 was not detected in spite of the flipped 3′-CMV promoter (Table 2), indicating that the efficient gene expression by the 3′-CMV promoter acts in the orientation-independent manner.

**Fig. 3:**
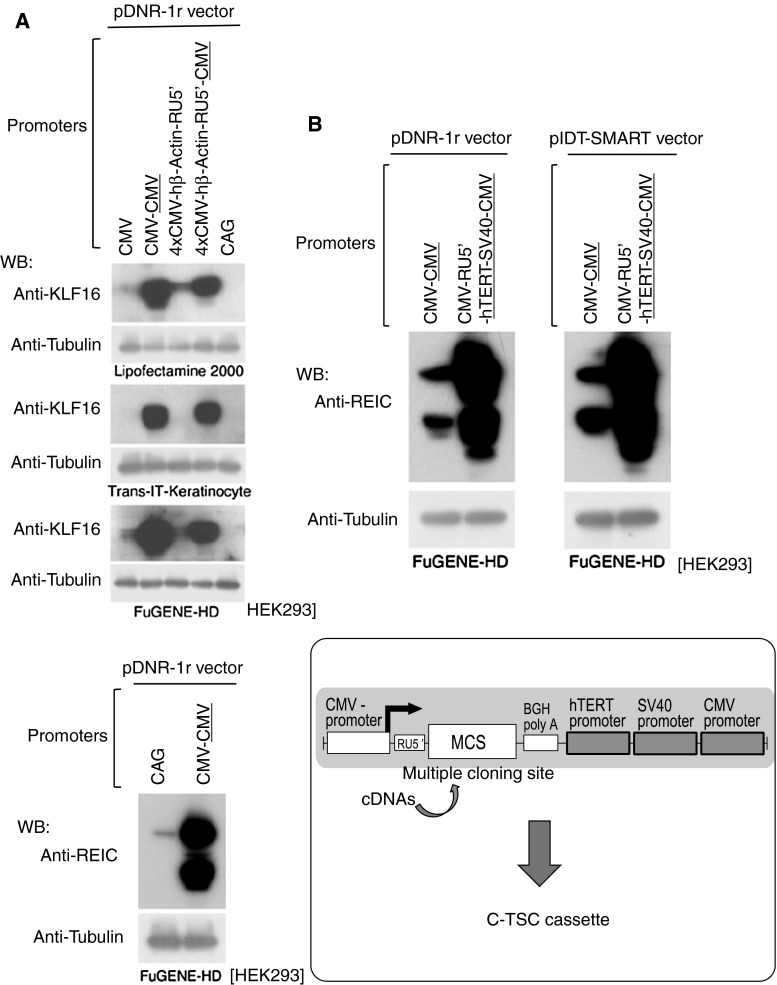
Construction of the C-TSC cassette. **a** Expression of KLF16 (*top*) or REIC/Dkk-3 (*bottom*) proteins after transfecting the indicated constructs including a vector containing the CAG promoter in HEK293 cells. Tubulin was used as a control for loaded amounts of protein. **b** To further improve the expression, 3 promoter sequences of hTERT, SV40, and CMV were tandemly inserted downstream of the REIC cDNA-BGH (bovine growth hormone)-polyA signal. RU5′, a part of the HTLV type 1 LTR inserted for better efficiency of transcription and translation. CMV–CMV and CMV–RU5′–hTERT–SV40–CMV were constructed on the basis of pDNR-1r and pIDT-SMART promoter-less vectors (*top*). The vectors were transfected to HEK293 cells, and levels of inserted REIC protein were determined by Western blot analysis (*top*). Tubulin was used as a control for loaded amounts of protein. Finally, we named the improved high expression cassette C-TSC (*bottom*)

The CAG promoter is one of the most potent promoters, and we therefore compared it with our vector constructs. As shown in Fig. 3a, vectors carrying the CMV promoter at each 3′-side of KLF16 (top) or REIC/Dkk-3 (bottom) cDNAs showed far higher expression levels than the conventional vector having the CAG promoter. To further improve the expression efficiency, we constructed 2 vectors carrying 3 promoters in tandem at the 3′-side, hTERT, SV40, and CMV promoters (Fig. 3b). Both vectors showed equally higher expression efficiency of REIC/Dkk-3 than the CMV–CMV vectors in HEK293 cells (Fig. 3b, top). Since pIDT-SMART (2,027 bp) is smaller in size and has a simpler structure than pDNR-1r (4,900 bp), we finally selected pIDT-SMART as the best basic vector to carry the high-level expression system, (5′) CMV promoter-RU5′-multiple cloning site (MCS)-BGH pA-(3′) hTERT–SV40–CMV promoters, named C-TSC cassette (Fig. 3b, bottom).

### Production of Erythropoietin (EPO) in Mammalian Cells

**Fig. 4:**
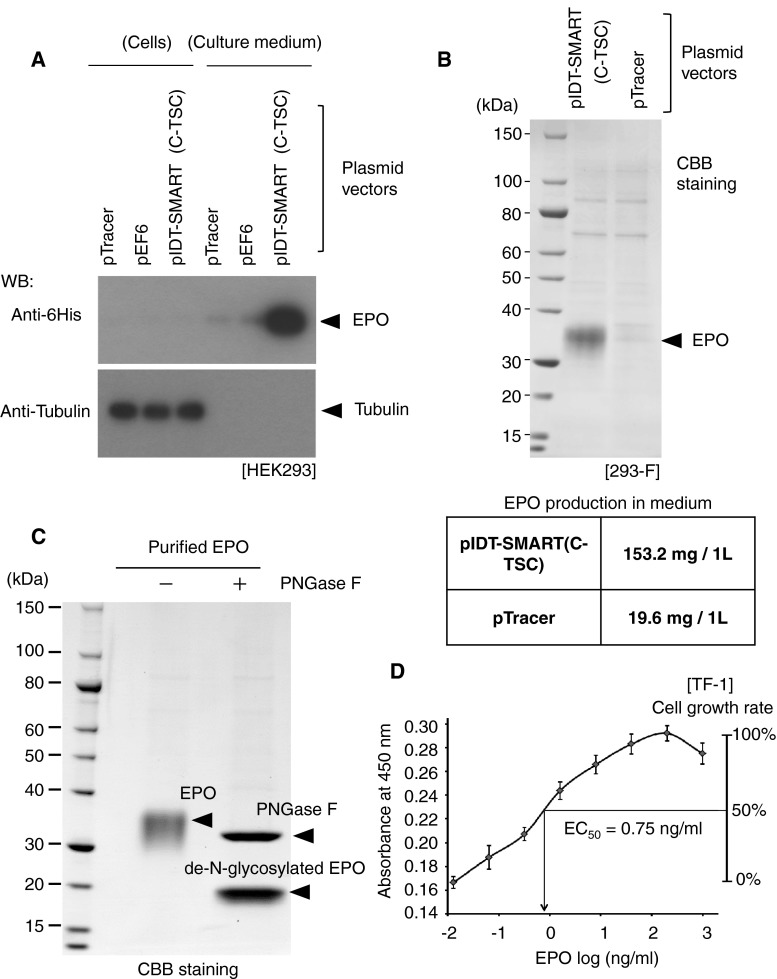
Expression levels of a secreted protein, erythropoietin (EPO). 24 h after transfection with the indicated vectors, 10 μl of serum-free culture medium and cellular protein extracts (10 μg) were subjected to electrophoresis, and EPO protein was detected by Western blot analysis. pTracer and pEF6 were from commercial source. **b** EPO was recovered from 1 liter of each culture medium of 293-F cells, electrophoresed, and stained with CBB (*top*). The amount of EPO obtained from 1 liter of each suspension culture (*bottom*). **c** The purified EPO was treated (+) or not treated (−) with PNGase F. **d** Effect of the purified EPO on growth of TF-1 cells was examined by an MTT assay

EPO is widely used for therapeutic purposes. It is a glycosylated protein and is, thus, produced in mammalian cells. We tested the possible use of the C-TSC cassette for efficient production of EPO. As shown in Fig. 4a, the use of pIDT-SMART (C-TSC) resulted in secretion of a large amount of EPO-6His into the medium of HEK293 cells compared with that in the case of conventional pTracer and pEF6. In a small-scale validation test in which 293-F cells were transfected with the vector, 153.2 mg/L of EPO-6His was recovered from the medium of cells transfected with pIDT-SMART (C-TSC), while only 19.6 mg/L of EPO-6His was recovered in the case of pTracer (Fig. 4b). The observed size of purified EPO-6His (30–40 kDa) was much larger than that estimated from the deduced amino acid sequence (mature EPO-6His without N-terminal signal sequence; 19.4 kDa). When the EPO-6His was treated with peptide-*N*-Glycosidase F (PNGase F), the size of the band was decreased to a size almost corresponding to the expected one (Fig. 4c), indicating that the purified EPO-6His is heavily glycosylated. Finally, we confirmed that the purified EPO-6His was biologically active (EC50 = 0.75 ng/ml) in vitro by measuring EPO-mediated growth induction of human erythroleukemic cell line, TF-1 (Fig. 4d).

### Possible Application of C-TSC Cassette for Gene Therapy

**Fig. 5:**
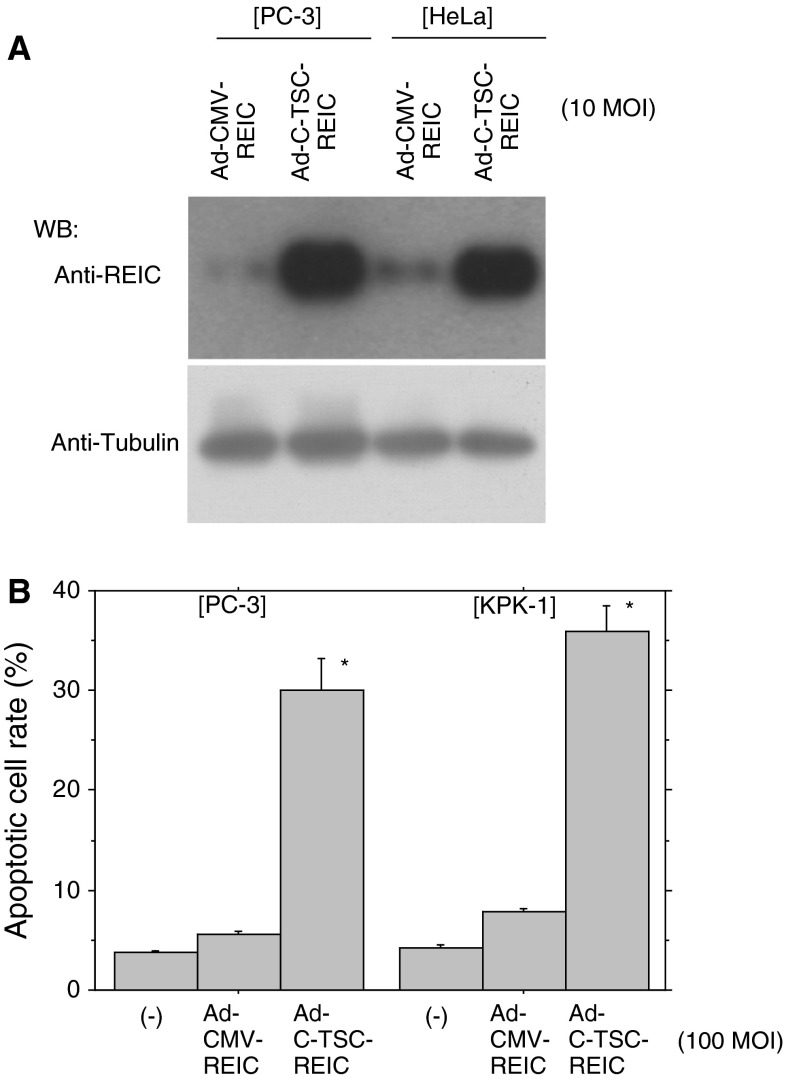
Expression of REIC/Dkk-3 by an adenovirus vector having the C-TSC cassette. **a** Expression of REIC/Dkk-3 was determined in human cancer cell lines (PC-3, prostate adenocarcinoma cell line; HeLa, cervix adenocarcinoma cell line). Tubulin was used as a control for loaded amounts of protein. **b** Induction of apoptosis in human cancer cell lines (PC-3; KPK-1, renal clear cell carcinoma cell line) by an adenovirus vectors carrying REIC/Dkk-3 infected at 100 MOI. * *p* < 0.05

We have been trying to develop a new gene therapeutic measure mainly against prostate cancer using an adenovirus carrying REIC/Dkk-3 cDNA [11, 13, 15–18]. Improvement of the expression efficiency may lead to better therapeutic achievements and less potential side effects. We, therefore, made an adenovirus vector having the C-TSC cassette and the cargo REIC/Dkk-3 cDNA. As shown in Fig. 5a, Ad-C-TSC showed remarkably higher expression levels of REIC/Dkk-3 than those by Ad-CMV in prostate cancer cells (PC-3 cells) and cervical cancer cells (HeLa cells). In accordance with this, infection of Ad-C-TSC-REIC efficiently induced apoptotic cell death in PC-3 cells and renal cancer cells (KPK-1 cells) (Fig. 5b).

## Discussion

In the present study, we developed a novel gene expression system that allows a cargo gene to be expressed with very high efficiency in various cell types. The essence of the expression system is to put three promoters, hTERT, SV40, and CMV promoters, in tandem downstream of the cargo gene. When combined with the 5′ CMV promoter, the observed expression levels of various cDNAs were far higher than those attained by the conventional CAG promoter, one of the strongest promoters for gene expression in mammalian cells [8]. Potent gene expression capability of our construct was observed not only in a plasmid construct but also in an adenovirus vector. The 3′-promoter was also active when combined with the SV40 promoter as well as the CMV promoter (data not shown). The mechanism by which insertion of promoters at the 3′-side of a cargo gene greatly enhances gene expression remains unclear. Cytoplasmic mRNA levels were much higher in the presence of 3′-CMV promoter that could be transcribed (Table 3). This may be due to efficient export of the mRNA and/or stabilization of the mRNA in the cytoplasm. Subsequently, we also detected the KLF16 protein at a remarkable level in cells transfected with the CMV–KLF16–CMV (flipped) (Fig. 2a). The expression level was comparable to that caused by the CMV–KLF16–CMV construct, suggesting that the 3′-CMV functions as an orientation-independent promoter in these experimental settings. Further studies are needed to clarify the detail mechanism of the extremely high level of proteins in the vector system.

REIC/Dkk-3 is a tumor suppressor gene and is significantly downregulated in a broad range of human cancer types [11, 13, 15]. We previously demonstrated that overexpression of REIC/Dkk-3 using a replication incompetent adenovirus vector (Ad-REIC) specifically induced apoptosis in various cancer cells [11, 13, 15–18]. Ad-C-TSC constructed on the basis of our findings showed higher expression of REIC/Dkk-3 and eventually induced apoptosis more efficiently at the same MOI (Fig. 5). Adenovirus vectors are presently used widely for practical therapeutics, and our Ad-C-TSC might therefore be a potent agent for such therapeutic approaches with less side effects.

Production of recombinant proteins in mammalian cells has many advantages compared with production in *E. coli* or other lower biological systems. When produced in mammalian cells, proteins are expected to be properly processed. These processes include cleavage and truncation, glycosylation, phosphorylation, and formation of disulfide bonds [19, 20]. Crystals of properly processed proteins are critically important for structural analysis, providing more precise information on functional proteins under physiological and functional conditions.

Recently, an increasing number of proteins, including peptide hormones, cytokines, and antibodies, have been used for therapy against various diseases [21, 22]. Production of such proteins with high efficiency determines the applicability. Many such protein agents have to be produced in mammalian cells and not in cells of bacteria or other lower animal origins, since properly processed proteins are often critical for exertion of the expected function. In a pilot experiment, we showed highly efficient production of EPO using pIDT-SMART (C-TSC), i.e., ~10 fold increase in efficiency compared with that in the case of a conventional pTracer vector (Fig. 4). Further optimization of vectors based on the present findings regarding the effect of the 3′ promoter may greatly contribute to applicability of protein therapeutics.
